# 

**Published:** 1877-09

**Authors:** 


### CONTENTS:

ARTICLE I.—The American Dental Association.................... 193
ARTICLE II.—A Canadian Dentist in Germany .................... 206
ARTICLE III—Remarks by Prof. Huxley to the London Dental Students.... ..... 215
ARTICLE IV.—Plastic Fillings. By Dr. C. L. Anderson.................... 221
ARTICLE V.—Alveolar Abscess. By J. A. Turner.................... 226
ARTICLE VI.—Chloroform and Ether in their Medico-Legal Relations. By Dr. A. M. Denig.................... 229

### EDITORIAL, ETC.

Letter from Oakland, Md. By H.................... 232

### MONTHLY SUMMARY.

The Cold Bath in Infantile Diarrhœa.................... 234

Carbolic Acid in Diarrhœa.................... 235

Croton Chloral in Dentistry.................... 235

Use of Tobacco by Students.................... 236

Death from Anæsthetics.................... 236

India Rubber Cloth in diseases of the Skin .................... 237

Apthæ.................... 237

Salicylic Acid Bad for Teeth.................... 237

Acidity of the Gastric Juice of Man.................... 238

Lacto-Phosphate of Lime as a Tooth Filling.................... 238

Therapeutic Use of Cold.................... 239

Osseous Lesions in Infantile Syphilis.................... 239

Dangerous Prescriptions.................... 240

Dr. Edward Warren.................... 240

